# Food consumption markers and sociodemographic factors associated with obesity among adult women registered in the Food and Nutrition Monitoring System (SISVAN) in Brazil: a cohort study, 2015–2019

**DOI:** 10.3389/fpubh.2024.1467339

**Published:** 2024-10-22

**Authors:** Anael Queirós Silva Barros, Italo Wesley Oliveira Aguiar, Lia Silveira Adriano, Antônio Augusto Ferreira Carioca, Dirce Maria Lobo Marchioni, Helena Alves de Carvalho Sampaio

**Affiliations:** ^1^Programa de Pós-Graduação em Saúde Coletiva, Universidade Estadual do Ceará, Fortaleza, Brazil; ^2^Programa de Pós-Graduação em Saúde Pública, Universidade Federal do Ceará, Fortaleza, Brazil; ^3^Curso de Nutrição, Universidade de Fortaleza, Fortaleza, Brazil; ^4^Departamento de Nutrição, Faculdade de Saúde Pública, Universidade de São Paulo, São Paulo, Brazil

**Keywords:** Food and Nutrition Monitoring, health information systems, cohort studies, nutritional status, obesity

## Abstract

**Introduction:**

Carrying out studies using data from Food and Nutritional Monitoring is crucial given the current epidemiological scenario. This scenario reveals a coexistence of malnutrition and nutritional deficiencies alongside overweight and obesity, particularly among adult women in Brazil. Analyzing the microdata generated by the system is essential for accurately representing food and nutrition indicators in the country.

**Objective:**

To analyze the association of food consumption markers and sociodemographic factors with changes in the Body Mass Index (BMI) among adult women.

**Methodology:**

A cohort study with a final sample of 30,354 women evaluated through individual records between the years 2015 and 2019. Nutritional status was assessed using body mass index (BMI). Food consumption markers used were the ones available in the system, which referred to foods consumed the previous day. Linear regression was used to evaluate factors associated with BMI and multiple Poisson regression was used to estimate the relative risk of obesity incidence.

**Results:**

There was an increase in BMI between 2015 and 2019. The average increase in BMI was lower in women who consumed, in the previous day, beans (β = −0,25; CI_95%_: −0.36; −0.13) and vegetables and/or legumes (β = −0.12; CI_95%_: −0.21; −0.04) and the increase was greater when they consumed hamburgers and/or sausages (β = 0.1; CI_95%_: 0.02; 0.19) adjusted by age, region and skin color. There was a 14% lower incidence of obesity in the group that consumed beans (RR = 0.86; 95% CI: 0.79; 0.93), and a 9% lower incidence when they consumed filled cookies, candies or treats (RR = 0.91; 95% CI 0.86; 0.96). The incidence of obesity was 11% higher in the group that consumed sweetened drinks (RR = 1.11; 95% CI: 1.05; 1.18) and 9% higher in the group that consumed instant noodles, packaged snacks or savory biscuits on the day before the 2015 registration (RR = 1.09; 95% CI: 1.03; 1.15).

**Conclusion:**

There was an increase in BMI in the population of adult women, rising nationally between 2015 and 2019, with the notable influence of diet on this change.

## 1 Introduction

The Brazilian Food and Nutrition Monitoring System (SISVAN) is a health information system, which functions as a technological tool for monitoring the food and nutritional situation of the Brazilian population receiving primary health care through the Unified Health System [*Sistema Único de Saúde*—SUS] (1).

It is worth highlighting its notoriety as a primary health care information system and a strong predictor of trends in the analysis of health conditions and the food and nutritional situation in Brazil. Thus, when it comes to adult individuals, especially women, the system becomes particularly useful. It also serves as a management tool for health professionals and managers, making it possible to monitor, manage, analyze and plan actions, supporting decision-making and priorities aimed at promoting and preventing health at a population level, improving the organization and care of nutritional care (2, 3).

The anthropometric assessment records of adult individuals assisted by the system are weight, height and waist circumference, where the cutoff points adopted for the Body Mass Index follow the recommendation of the World Health Organization (4). The index is calculated by dividing the weight in kilograms (kg) by the height in meters (m) squared (kg/m^2^), which becomes a satisfactory tool for predicting population risks (5). Food consumption markers assess foods that were consumed the previous day, identifying healthy or unhealthy behaviors, according to the recommendations of the food guide for the Brazilian population (6). There is a notable increase in BMI, which confirms the nutritional changes experienced in Brazil and around the world, with a decline in the prevalence of malnutrition and an increase in obesity and overweight individuals (7), and it is object of study among Brazilian adults (8), based on a comprehensive set of surveys between 1975 and 2019, exposing, over time, the classic “X” shape with the nutritional transition being greater and more representative among women, pointing to a decrease in underweight rates among men and women, inversely to the increase in the obesity trajectory among both sexes over the years.

Despite the relevance of the subject, this is the first Brazilian cohort study to date to analyze the association of food consumption markers and sociodemographic factors associated with obesity among adult women, using microdata from the Food and Nutrition Surveillance System, assessing the representativeness of the food and nutrition indicators produced by the system. Thus, the importance of conducting studies with data from this system is highlighted, especially considering the epidemiological scenario, which indicates a simultaneity between malnutrition and nutritional deficiencies, overweight and obesity in the adult population in general (9–11) and especially in women (12). Therefore, the objective of this study was to analyze the association of food consumption markers and sociodemographic factors associated with obesity among adult women.

## 2 Methodology

### 2.1 Study design

This is an observational epidemiological study using an historical cohort of adult women from the recorded data on nutritional status and markers of food consumption of SISVAN at a national level, in the period between 2015 and 2019. Cohort studies are important for observing, in a previously defined sample, the incidence of a given disease or phenomenon related to health or illness in a temporal sequence of events over time. This type of study thus allows the identification of possible risk factors, understanding the development of diseases and supporting decisions in public health policies, in addition to serving as a basis for future studies (13). Cohort studies have been considered the most reliable, if not the only method for studying the incidence and natural history of diseases (14, 15). The choice of this period is due to the fact that 2015 was when the new food consumption forms were introduced, and 2019 was the last year before the start of the COVID-19 pandemic. In 2019, no measures to control and prevent the disease had been implemented. Since these measures influenced daily habits and regular access to health services, it is assumed that the data for the year used would represent the usual profile of the target population.

The research complied with the standards of the National Health Council and instructions of regulation number 884, December 13, 2011 (16), on the transfer of data contained in the national databases of information systems managed by the Health Care Department and the Brazilian Health Ministry. The study project was approved by the Human Research Ethics Committee of the University of Fortaleza (UNIFOR) on October 20, 2020 with protocol no. 4,348,452, and Certificate of Presentation for Ethical Assessment n° 31540320.9.1001.5052.

### 2.2 Context

The research analyzed data from adult women from all over Brazil, identified through the National Health Card in the Food and Nutrition Surveillance System (SISVAN), with the analysis units being users of Primary Health Care whose anthropometric records were carried out in 2015 and then in 2019 in their macro-regions (North; Northeast; South; Southeast; Midwest) and Federation Units (FUs) (17).

The data analyzed come from SISVAN, provided by the Ministry of Health, as requested from the General Coordination of Food and Nutrition, the microdata provided are composed of monitoring records from SISVAN itself, its Web version which also includes the migration of data from the *Bolsa Fam*í*lia* Program; and through the citizen's electronic medical record and simplified data collection software, and the integration of SISVAN with the e-SUS Primary Care Strategy, where the migration of existing data in the Primary Care System (SISAB) to SISVAN database, according to a technical note from the Ministry of Health (18).

### 2.3 Participants

**Figure 1:**
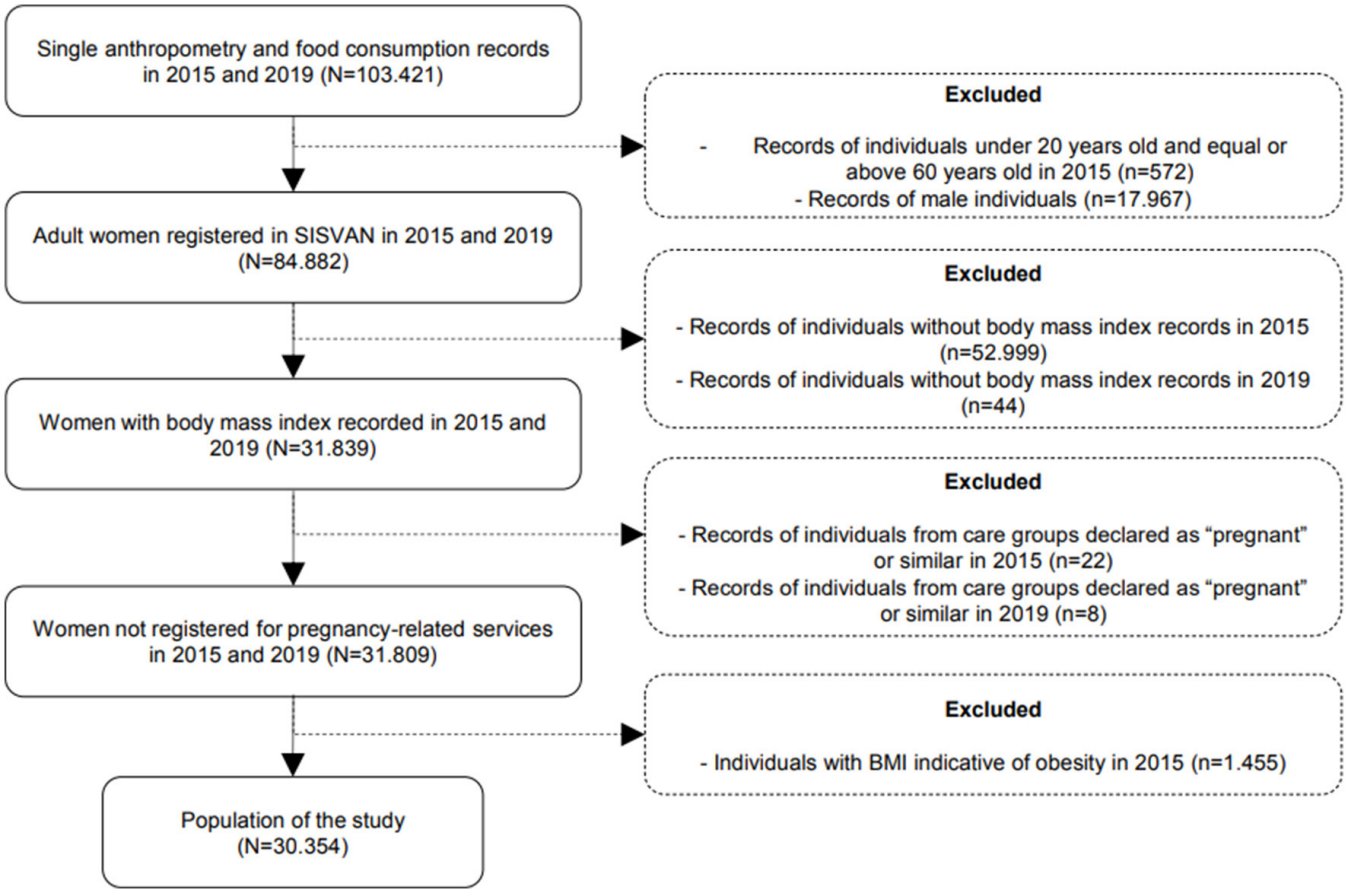
Flowchart description for obtaining the final research sample (*N* = 30,354). Brazil, 2015 and 2019. Source: created by the authors.

The inclusion criteria for this cohort study were, cumulatively: (a) registered in SISVAN, being female, aged between 20 and 59 years in 2015; (b) records of the same individuals in 2019; and (c) the completion of data regarding anthropometry and food consumption in the years 2015 and 2019. We considered exclusion criteria to be: (a) records linked to care programs for pregnant women; (b) duplicate records (food consumption and anthropometry recorded more than once a year), with only records of the last assessment for 2015 and 2019 being kept; (c) biologically implausible values with weight < 30 and ≥300 kg (19); and (d) observations with a height < 1.20 and ≥2.20 m (19). When verifying the incidence of obesity, an additional exclusion criterion was the individual having a BMI ≥ 30 kg/m^2^ in 2015, the year of the cohort's baseline. The sample consisted of 84,882 women followed up in PHC between 2015 and 2019. After filters and exclusions, and considering only the cases that were present in the databases, the final sample was 30,354 women. The process for obtaining the sample is represented in Figure 1.

### 2.4 Variables

Demographic, socioeconomic, food, and nutritional variables were used to characterize and analyze the sample. The demographic and socioeconomic variables used in this study were: geographic region (categorized as North; Northeast; Southeast; South; Central-West), age group (20–29; 30–39; 40–49; 50–59 years), and race/color (white; black; mixed-race; Asian; indigenous) (17).

The classification of nutritional status used by SISVAN is according to the Body Mass Index (BMI), in units of kg/m^2^. The cutoff points for BMI classification follow the World Health Organization (4) recommendation: obesity, when BMI ≥ 30 kg/m^2^.

Food consumption markers were collected using a screening form (20) proposed by seven markers, where the foods consumed during the previous day are noted, using an available list, with “yes,” “no,” or “don't know” response options. This enables the recognition of intake related to healthy or unhealthy eating, based on the proposal of the Food Guide for the Brazilian Population (6).

In line with the Food Guide for the Brazilian Population (6), the markers that characterize a healthy diet are: beans; fresh fruit; vegetables and/or legumes; and unhealthy diet are: hamburgers and/or sausages; sweetened drinks; instant noodles, packaged snacks or savory biscuits; and filled cookies, sweets, or treats (21).

### 2.5 Data source

The information used for this research came from the General Coordination of Food and Nutrition through the Ministry of Health. The data was organized in an Excel^®^ spreadsheet in October 2022. The R Studio software was used for the process of filters and exclusions, to finalize the final database.

### 2.6 Data analysis

The variable “age group” was calculated based on age categorization, in complete years. The individual difference in BMI was calculated for each participant, based on the BMI value obtained in 2019 subtracted from the BMI value assessed in 2015. The frequency of BMI differences was observed by creating a histogram, which indicated a normal distribution.

The sociodemographic and anthropometric aspects of the sample were characterized using absolute and relative frequency. The average difference in BMI between the studied groups was calculated, and its association with sociodemographic aspects and food consumption was verified. The unadjusted association was estimated using simple linear regression, while the association adjusted for the sociodemographic variables of age, region and skin color was estimated using multiple linear regression. For the linear regression model, the variation in BMI was considered the outcome and the sociodemographic and food consumption aspects were considered exposure.

Food consumption markers were considered the exposure (collected in 2015) and the diagnosis of obesity was considered outcome (collected in 2019). It is noteworthy that cases of obesity prevalence were excluded in 2015.

The incidence rate of obesity was calculated by dividing the new cases in 2019 by the number of individuals at risk in 2015, multiplied by 100.

The incidence of obesity was compared between the groups using the relative risk estimate and its respective 95% confidence interval, obtained by multiple Poisson regression, adjusted for age, region and skin color, with a robust variance estimator. The analyses were carried out at a two-tailed α level of 0.05, with the aid of the Stata Statistical: version 11.2 (College Station, TX: StataCorp LLC).

## 3 Results

In the macro-regions, it was observed that the Southeastern region (β = −0.18; 95% CI: −0.28; −0.09) showed a lower increase in BMI when compared to the Northeast, while the opposite occurred in the Central-Western region (β = 0.28; 95% CI: 0.13; 0.42). The increase in BMI occurred in the age groups of 30–39 years (β = −0.3; 95% CI: −0.38; −0.21), 40–49 (β = −0.45; 95% CI: −0.56; −0.34), and 50–59 years (β = −0.69; 95% CI: −0.84; −0.54), compared to the age group of 20–29 years (Table 1).

**Table 1:** Average difference in BMI units (kg/m^2^) and association with demographic and socioeconomic aspects among adult women registered in SISVAN (*n* = 30.354), Brazil, 2015–2019.

**Characteristic^a^**	***n* (%)^a^**	**Δx BMI^b^**	**β^c^**	**Confidence interval (CI_95%_)**
**Region**				
Northeast	12,674 (41.8)	1.23	Ref.	
Southeast	7,802 (25.7)	1.04	−0.18	**(−0.28;** **−0.09)**
North	6,022 (19.8)	1.3	0.08	(−0.02; 0.18)
Center-west	2,459 (8.1)	1.5	0.28	**(0.13; 0.42)**
South	1,397 (4.6)	1.32	0.09	(−0.09; 0.28)
**Age group in 2015 (years)**				
20–29	1,3348 (44.0)	1.44	Ref.	
30–39	9,624 (31.7)	1.15	−0.3	**(−0.38;** **−0.21)**
40–49	5,053 (16.7)	0.99	−0.45	**(−0.56;** **−0.34)**
50–59	2,329 (7.7)	0.75	−0.69	**(−0.84;** **−0.54)**
**Race or skin color**				
White	6,454 (21.3)	1.18	Ref.	
Mixed-race	13,399 (44.1)	1.26	0.08	(−0.02; 0.18)
Black	1,932 (6.4)	1.31	0.13	(−0.04; 0.30)
Asian	3,170 (10.4)	1.16	−0.02	(−0.17; 0.12)
Indigenous	230 (0.8)	0.97	−0.21	(−0.65; 0.23)
No information	5,169 (17.0)	1.2	0.02	(−0.10; 0.15)

^a^Column percentage, consisting of the absolute and relative frequency of each category.

^b^Average difference in body mass index (BMI) measured in kg/m^2^, between 2015 and 2019.

^c^Beta coefficients (β) and 95% confidence intervals (95% CI) obtained by simple linear regression, with the dependent variable being the average difference in BMI between 2015 and 2019.

Values in bold were statistically significant.

Source: created by the authors.

The average increase in BMI was lower in those who reported consuming beans the previous day (β = −0.25; 95% CI: −0.36; −0.13) and vegetables (β = −0. 12; 95% CI: −0.21; −0.04) and higher when consuming hamburgers and/or sausages (β = 0.1; 95% CI: 0.02; 0.19) adjusted by age, region and color of skin (Table 2).

**Table 2:** Average difference in BMI units (kg/m^2^) and association with markers of food consumption among adult women registered in SISVAN (*n* = 30.354), Brazil, 2015–2019.

**Characteristic^a^**	***n* (%)^a^**	**Δx BMI^b^**	**Unadjusted association** ^**c**^		**Age-adjusted association** ^**d**^		**Association adjusted by age, region, and skin color** ^**e**^	
			β	**(CI**_95%_ **)**	β	**(IC**_95%_ **)**	β	**(IC**_95%_ **)**
**Eat beans** ^*^								
No	3,666 (12.1)	1.47	ref.		ref.		ref.	
Yes	26,395 (87.0)	1.19	−0.28	**(−0.39;** **−0.16)**	−0.26	**(−0.37;** **−0.14)**	−0.25	**(−0.36;** **−0.13)**
Don't know	293 (1.0)	1.11	−0.36	(−0.76; 0.04)	−0.41	**(−0.81;** **−0.01)**	−0.26	(−0.66; 0.14)
**Eat fresh fruit** ^*^								
No	8,166 (26.9)	1.28	ref.		ref.		ref.	
Yes	21,878 (72.1)	1.2	−0.09	(−0.17; 0.00)	−0.09	(−0.17; 0.00)	−0.08	(−0.17; 0.00)
Don't know	310 (1.0)	1.4	0.12	(−0.26; 0.5)	0.05	(−0.33; 0.43)	0.2	(−0.18; 0.58)
**Eat vegetables and/or legumes** ^*^								
No	8,559 (28.2)	1.32	ref.		ref.		ref.	
Yes	21,460 (70.7)	1.18	−0.14	**(−0.23;** **−0.06)**	−0.13	**(−0.22;** **−0.05)**	−0.12	(–**0.21;** **−0.04)**
Don't know	335 (1.1)	1.16	−0.16	(−0.53; 0.20)	−0.23	(−0.59; 0.14)	−0.1	(−0.47; 0.26)
**Eat hamburgers and/or sausages** ^*^								
No	20,929 (69.0)	1.18	ref.		ref.		ref.	
Yes	8,896 (29.3)	1.31	0.13	(0.05; 0.22)	0.11	**(0.03; 0.19)**	0.1	**(0.02; 0.19)**
Don't know	529 (1.7)	1.33	0.15	(−0.13; 0.44)	0.1	(−0.19; 0.39)	0.17	(−0.12; 0.46)
**Drink sweetened drinks** ^*^								
No	12,279 (40.5)	1.19	ref.		ref.		ref.	
Yes	17,574 (57.9)	1.24	0.05	(−0.02; 0.13)	0.02	(−0.06; 0.10)	0.03	(−0.05; 0.11)
Don't know	501 (1.65)	1.46	0.27	(−0.03; 0.57)	0.21	(−0.09; 0.51)	0.28	(−0.02; 0.58)
**Eat instant noodles. packaged snacks or savory biscuits** ^*^								
No	17,780 (58.58)	1.19	ref.		ref.		ref.	
Yes	12,024 (39.6)	1.26	0.06	(−0.02; 0.14)	0.05	(−0.03; 0.12)	0.03	(−0.05; 0.11)
Don't know	550 (1.8)	1.38	0.18	(−0.10; 0.46)	0.14	(−0.15; 0.42)	0.18	(−0.10; 0.46)
**Eat filled cookies. candies or treats** ^*^								
No	18,693 (61.6)	1.2	ref.		ref.		ref.	
Yes	11,115 (36.6)	1.26	0.06	(−0.02; 0.14)	0.01	(−0.07; 0.08)	0.00	(−0.08; 0.08)
Don't know	546 (1.8)	1.27	0.07	(−0.21; 0.36)	0.02	(−0.27; 0.30)	0.07	(−0.21; 0.36)

^a^Column percentage, consisting of the absolute and relative frequency of each category.

^b^Average difference in body mass index (BMI) measured in kg/m^2^ between 2015 and 2019.

^c^Beta coefficients (β) and 95% confidence intervals (95% CI) obtained by simple linear regression, with the average difference in BMI between 2015 and 2019 as the dependent variable.

^d^Beta coefficients (β) and 95% confidence intervals (95% CI) obtained by multiple linear regression, adjusted for age (in 2015), with the average difference in BMI between 2015 and 2019 as the dependent variable.

^e^Beta coefficients (β) and 95% confidence intervals (95%CI) obtained by multiple linear regression. Adjusted for age, region, color, and education (in 2015), with the dependent variable being the average difference in BMI between 2015 and 2019.

^*^Referring to consumption from the previous day. Values in bold were statistically significant.

Source: created by the authors.

The incidence of obesity (BMI ≥ 30 kg/m^2^) and association with sociodemographic aspects among adult women (20–59 years old) registered in SISVAN was 11% lower in the Northern region (RR = 0.89; 95% CI: 0.82; 0.96) and 17% higher in the Southern region (RR = 1.17; 95% CI: 1.03; 1.32) when compared to the Northeastern region. Compared to the age group of 20–29 years, the risk of obesity was 20% higher in the age group between 40 and 49 years (RR = 1.28; 95% CI: 1.20; 1.37) and 32% lower in the age group aged 50–59 years (RR = 0.68; 95% CI: 0.60; 0.78). The incidence of obesity was 13% lower in Asian people (RR = 0.87; 95% CI: 0.78; 0.97) when compared to white people (Table 3).

**Table 3:** Incidence of obesity (BMI ≥ 30 kg/m^2^) and association with sociodemographic aspects among adult women registered in SISVAN (*n* = 30.354), Brazil, 2015–2019.

**Characteristic^a^**	***n* (%)^a^**	**New cases** ^**b**^		**Relative risk RR^c^**	**Confidence interval (CI_95%_)**
		**Count** ^b^	**Incidence ratio (%)**		
**Region**					
Northeast	12,674 (41.8)	1.905	15,0	1	
Southeast	7,802 (25.7)	1.188	15,2	1.01	(0.95; 1.08)
North	6,022 (19.8)	805	13,4	0.89	**(0.82; 0.96)**
Center-West	2,459 (8.1)	404	16,4	1.09	(0.99; 1.21)
South	1,397 (4.6)	245	17,5	1.17	**(1.03; 1.32)**
**Age group in 2015 (years)**					
20–29	13,348 (44.0)	1.994	14,9	1	
30–39	9,624 (31.7)	1.347	14,0	0.94	(0.88; 1.00)
40–49	5,053 (16.7)	968	19,2	1.28	**(1.20; 1.37)**
50–59	2,329 (7.7)	238	10,2	0.68	**(0.60; 0.78)**
**Race or skin color**					
White	6,454 (21.3)	978	15,2	1	
Mixed-race	13,399 (44.1)	2.120	15,8	1.04	(0.97; 1.12)
Black	1,932 (6.4)	295	15,3	1.01	(0.89; 1.14)
Asian	3,170 (10.4)	417	13,2	0.87	**(0.78; 0.97)**
Indigenous	230 (0.8)	24	10,4	0.69	(0.47; 1.01)
No information	5,169 (17.0)	713	13,8	0.91	(0.83; 1.00)

^a^Column percentage, consisting of the absolute and relative frequency of each category.

^b^Count of new cases and incidence proportion, with relative frequency of new cases given the total number of individuals at risk in each category.

^c^Relative risks (RR) and 95% confidence intervals (95% CI) obtained by simple Poisson regression with robust variance estimator, with new cases of obesity as the dependent variable between 2015 and 2019. Values in bold were statistically significant.

Source: created by the authors.

Regarding the incidence of obesity (BMI ≥ 30 kg/m^2^) and association with markers of food consumption among adult women aged 20–59 years registered in SISVAN, regardless of age, region, and skin color, we observed a 14% lower incidence when individuals said they ate breakfast the day before, compared to individuals who did not consume a breakfast (RR = 0.83; 95% CI: 0.73; 0.95). There was also a 15% lower incidence of obesity in the group that consumed a morning snack (RR = 0.85; 95% CI: 0.80; 0.90), 28% lower in the group that ate lunch (RR = 0.72; 95% CI: 0.58; 0.89), 19% lower in the group that had an afternoon snack (RR = 0.81; 95% CI: 0.76; 0.87) and 10% lower in the group that had an evening meal (RR = 0.9; 95% CI 0.83; 0.98). A 14% lower incidence of obesity was found in the group that consumed beans (RR = 0.86; 95% CI: 0.79; 0.93), and 9% lower when individuals said they consumed filled cookies, candies or treats (RR = 0.91; 95% CI: 0.86; 0.96), 11% higher in the group that consumed sweetened drinks (RR = 1.11; 95% CI: 1.05; 1.18) and 9% higher in the group who consumed instant noodles, packaged snacks or savory biscuits on the day before the 2015 records (RR = 1.09; 95% CI: 1.03; 1.15). In regard to the consumption of vegetables and/or legumes, we observed 7% higher risk of obesity in the unadjusted association (RR = 1.07; 95% CI: 1.01; 1.14) and 7% in the age-adjusted association (RR = 1.07; 95% CI: 1.01; 1.14), however, when analyzed, the association adjusted for age, region and skin color did not appear to be statistically significantly associated (RR = 1.06; 95% CI: 1.00; 1.13; Table 4).

**Table 4:** Incidence of obesity (BMI ≥ 30 kg/m^2^) and association with food consumption markers among adult women registered in SISVAN (*n* = 30.354), Brazil, 2015–2019.

		**New cases** ^**b**^		**Unadjusted association** ^**c**^		**Age-adjusted association** ^**d**^		**Association adjusted by age. region and skin color** ^**e**^	

**Characteristic**	***n*** **(%)**^a^	**Count. (** ***n*** **)**	**Incidence ratio (%)**	**Relative risk (RR)**	**Confidence interval (CI**_95%_ **)**	**Relative risk (RR)**	**Confidence interval (CI**_95%_ **)**	**Relative risk (RR)**	**Confidence interval (CI**_95%_ **)**
**Eat beans** ^*^									
No	3,666 (12.08)	618	16,9	1		1		1	
Yes	26,395 (86.96)	3.893	14,8	0.87	(0.81; 0.95)	0.87	**(0.81; 0.94)**	0.86	**(0.79; 0.93)**
**Eat fresh fruit** ^*^									
No	8,166 (26.9)	1.186	14,5	1		1		1	
Yes	21,878 (72.08)	3.319	15,2	1.04	(0.98; 1.11)	1.04	(0.98; 1.11)	1.04	(0.98; 1.11)
**Eat vegetables and/or legumes** ^*^									
No	8,559 (28.2)	1.221	14,3	1		1		1	
Yes	21,460 (70.7)	3.288	15,3	1.07	**(1.01; 1.14)**	1.07	**(1.01; 1.14)**	1.06	(1.00; 1.13)
**Eat hamburgers and/or sausages** ^*^									
No	20,929 (68.95)	3.156	15,1	1		1		1	
Yes	8,896 (29.31)	1.324	14,9	0.99	(0.93; 1.05)	0.99	(0.93; 1.05)	0.98	(0.92; 1.04)
**Drink sweetened drinks** ^*^									
No	12,279 (40.45)	1.725	14,1	1		1		1	
Yes	17,574 (57.9)	2.745	15,6	1.11	**(1.05; 1.18)**	1.11	**(1.05; 1.18)**	1.11	**(1.05; 1.18)**
**Eat instant noodles. packaged snacks or savory biscuits** ^*^									
No	17,780 (58.58)	2.574	14,5	1		1		1	
Yes	12,024 (39.61)	1.898	15,8	1.09	**(1.03; 1.15)**	1.09	**(1.03; 1.15)**	1.09	**(1.03; 1.15)**
**Eat filled cookies. candies or treats** ^*^									
No	18,693 (61.58)	2.902	15,5	1		1		1	
Yes	11,115 (36.62)	1.571	14,1	0.91	**(0.86; 0.96)**	0.91	**(0.86; 0.96)**	0.91	**(0.86; 0.96)**

^a^Column percentage, consisting of the absolute and relative frequency of each category.

^b^Count of new cases and incidence proportion, with relative frequency of new cases given the total number of individuals at risk in each category.

^c^Relative risks (RR) and 95% confidence intervals (95% CI) obtained by simple Poisson regression with robust variance estimator, with new cases of obesity between 2015 and 2019 as the dependent variable.

^d^Relative risks (RR) and 95% confidence intervals (95% CI) obtained by multiple Poisson regression with robust variance estimator, using new cases of obesity between 2015 and 2019 as the dependent variable, adjusted for age.

^e^Relative risks (RR) and 95% confidence intervals (95% CI) obtained by multiple Poisson regression with robust variance estimator, with new cases of obesity as the dependent variable between 2015 and 2019, adjusted by age, region, and color of skin.

^*^Referring to consumption from the previous day. Values in bold were statistically significant.

Source: created by the authors.

## 4 Discussion

In this cohort study, we identified sociodemographic factors and markers of food consumption associated with BMI and obesity. There was an increase in BMI between 2015 and 2019, which varied between regions and age groups. There was a lower average increase in BMI and a lower incidence of obesity in women who reported eating, in the previous day, foods like beans, vegetables, and/or legumes. There was a greater increase in BMI when they consumed hamburgers and/or sausages. The incidence of obesity was lower with the consumption of beans and filled cookies, candies or treats and higher among women who reported consuming sweetened drinks, instant noodles, packaged snacks or savory biscuits.

When analyzing the increase in BMI of the population of Brazilian adult women in association with demographic aspects, we observed that the Southeastern region showed a lower increase in BMI, compared to the Northeastern region, while the opposite occurred in the Central-Western region. Thus, the Central-Western region leads the increase in an overweight and obesity population in the period analyzed. Regarding this finding, the literature actually provides data on the increased prevalence of being overweight and obesity in this region (22, 23). With reference to the incidence of obesity and its association with sociodemographic aspects, we found a lower incidence in the Northern region and a higher incidence in the Southern region, when compared to the Northeastern region. It is noteworthy that nutritional status can be considered an indicator of health and quality of life, reflecting a development model; thus, the different socioeconomic realities in the Brazilian territory are reflected in the nutritional status, which varies between regions of the country (9, 10).

The increase in BMI over the years in the present study is in line with findings from other nationwide research (8, 19). Therefore, the pattern of obesity in Brazil does not affect the population in a random or homogeneous manner, it tends to vary according to gender and socioeconomic status, as in other developing countries, with a higher prevalence of obesity among women (24, 25). Silva et al. (26) emphasize that women are more likely to experience higher levels of body weight, when these factors are directly associated with conditions such as income, marital status, physical activity, consumption of beans, consumption of alcoholic beverages, and smoking cigarettes, as these are all variables that have a greater relationship with gender inequality.

The number of data records in SISVAN also varied by macro-region. We found evidence that the Northeast presented a greater number of registered records of adult women who were attended to in PHC by the Unified Health System (SUS), in agreement with national studies, indicating improvement in the monitoring of nutritional status over the years in this region (10, 22, 27). This may be due to the monitoring of beneficiary families of the *Bolsa Fam*í*lia* program, with a greater number of records and monitoring in this region, as this program has greater coverage in the northeast region, due to the presence of higher poverty rates (28).

Despite the consumption of basic and traditional foods in the Brazilian diet, considered healthy markers, there is a high intake of foods with high energy value and low nutritional value, such as processed and ready-to-eat foods, contributing to the increase in being overweight and obesity in the population (29). Data found in this research, which have already been described in the literature, show an association between high consumption of sweetened and ultra-processed drinks, with the incidence of obesity and increased risk of NCDs (30, 31).

According to the Family Budget Survey, the acquisition of these foods rose from 12% in the 2002–2003 (32) survey to 16% in the 2007–2008 (33) survey and then 18.4% in 2017–2018 (34). Ultra-processed food consumption was responsible for ~57,000 premature deaths in Brazil or 10.5% of all premature deaths in adults aged 30–69 in Brazil (29, 35).

The results presented allowed us to identify healthy and unhealthy markers, as well as eating habits of women in Brazil monitored by SISVAN. Thus, we observed a lower increase in BMI in women who reported the habit of consuming at least five meals a day, including breakfast, morning snack, lunch, afternoon snack and evening meal, as well as consuming beans and vegetables and/or legumes the day before, and a higher increase when they consumed hamburgers and/or sausages. People who seem to split their meals have a regular supply of energy, maintenance of blood glucose levels and stabilization of metabolism, even when maintaining or reducing weight (36).

The incidence of obesity was lower with the consumption of beans and filled cookies, candies or treats and higher among women who reported consuming sweetened drinks, instant noodles, packaged snacks, or savory biscuits. We highlight in this research some unexpected findings regarding the incidence of obesity associated with the consumption of vegetables and/or legumes, where we observed a higher relative risk of the incidence of obesity when analyzed by unadjusted association and with the association adjusted by age, however, when we analyzed the association adjusted for age, region and skin color, we did not observe an association, while in regard to fruit intake, which should have some positive association with protective factors in the study, there was no significance in the analyses. Therefore, a possible justification would perhaps be a discrepancy caused by the lower prevalence of intake of these foods.

The intake of filled cookies, candies or treats, which was considered protective against obesity, was not expected. Some aspects should be discussed in relation to this finding. Firstly, the type of instrument used, which does not investigate food quantities consumed. It is possible that to detect the consumption of high amounts of this food group would be associated with a higher incidence of obesity. It should also be noted that within this food group, filled cookies may not necessarily be harmful, depending on the quantity and type of cookie, as the food industry has long been trying to end the impression that cookies are a villain of health and sell more of these products, reducing the presence of unhealthy ingredients and adding healthy ones (37, 38). Thus, the consumption of cookies has increased over the years and new production lines are emerging in the Brazilian food products market, which vary according to consumer demand, and include cookies with low fat content, added vitamins and minerals, with fiber, without gluten, are organic, lactose-free, and sugar-free, among others (28).

Likewise, it is important to point out that there is the consumption of beans, which emerges as a protective factor and their presence can counterbalance the harmful effects of some foods, in the case found here, filled cookies, candies and treats. Thus, the highest food frequencies, according to the 2008–2009 Family Budget Survey (33) and the 2017–2018 Family Budget Survey (34), which include rice and beans, show that Brazilians maintain a dietary pattern based on these two foods. Similar results on the consumption of beans as a marker of healthy eating in the Brazilian adult population were observed in previous population surveys (39, 40).

Another limitation attributed to the instrument used is that, in addition to not investigating quantity consumed, it does not investigate habituality of consumption, restricting itself to consumption from the previous day, which may prevent detecting other findings relating to the effects of different food groups on the BMI and the incidence of obesity. There is a need to delve deeper into the unexpected occurrences detected here, taking into account the limitations highlighted, in order to have a complete picture of the factors influencing BMI and the incidence of obesity. Although a quantitative investigation within the instrument's proposal seems unfeasible, it is necessary to think about possible strategies in order to improve the quality of research results that use this instrument.

From another perspective, Lourenço et al. (41) characterized the internal structure of the SISVAN form and analyzed the evidence of measurement invariance between Brazilian macro-regions, stages of life and over time. In this way, the authors clarify that the internal structure of the form adequately reflects its conceptual basis, with the stability of factors related to healthy and unhealthy eating in configuration, loads and scale in the sections investigated, enhancing its use in research in the SUS.

Given the results found, the coverage of the population-level dimension that assesses the markers related to healthy or unhealthy nutrition is highlighted, and enables food and nutritional monitoring and vigilance (42). The Food Guide for the Brazilian Population (6) provides information and recommendations on foods, meals and eating practices, based on the NOVA Classification, encouraging the intake of fresh and minimally processed foods.

This study has strengths and some limitations. Regarding its strengths, SISVAN represents the main information system at the interface with Food and Nutrition Security, being part of the third guideline of the National Food and Nutrition Policy (42). It is worth highlighting its reliability as a Primary Health Care information system and a strong predictor of trends in the analysis of health conditions and the food and nutritional situation in Brazil. It also serves as a management tool for health professionals and managers, improving the organization and care of nutritional care (1, 17).

SISVAN has been improved and updated over the years, improving its coverage and data quality, favoring early diagnosis, decision-making and the formulation and/or reformulation of public policies and health promotion actions in the APS, aimed at the country's food and nutritional needs (9, 22). It is important to highlight that the information present in this study's database is national data from basic health units in Brazil, which were collected using a standardized instrument recommended by the Ministry of Health (MS). Furthermore, the questions are objective and clear, and easy to apply by Primary Health Care professionals (41).

Among the limitations of this study, it is important to note the need for caution when generalizing the findings to the population of Brazilian women as a whole, since the sample represented by the SISVAN user population has peculiar characteristics, closer to the population benefiting from social programs. In addition, it is mentioned that the marker form is subject to recall biases or influenced by the fact that the interviewees are aware of socially accepted behaviors. There may also be failures in obtaining the data. Another limitation of the study lies in eating habits, especially when the previous day's consumption may be atypical, such as after holidays and weekends, where the eating routine is usually null. Therefore, the use of secondary data from SISVAN, which in turn comes from different sources, does not rule out the possibility of underreporting bias or errors in collection, filling in and typing in information management.

## 5 Conclusion

There was an increase in BMI in the population of adult women, rising nationally in the five national macro-regions in Brazil, between 2015 and 2019. The influence of diet on this evolution was noted. There was less variation in BMI among women who reported consuming beans, vegetables and/or legumes. The incidence of obesity was lower among those who consumed beans and filled cookies, candies or treats, and higher among those who consumed sweetened drinks, instant noodles, packaged snacks or savory biscuits.

Thus, our findings highlight SISVAN as a tool that facilitates the operationalization of a large database for Food and Nutrition Surveillance in Primary Health Care, and can support the implementation of similar studies, despite the various challenges reported in the literature over the years for its use, such as supply, use and coverage. Even so, the system has advanced, both in terms of its coverage, data quality and the formulation of policies and programs using its data. Finally, the need for more studies using data from this system on the nutritional status and food consumption of women treated in PHC in Brazil is highlighted.

## Data Availability

The original contributions presented in the study are included in the article/supplementary material, further inquiries can be directed to the corresponding author.
